# Impact of warming blood transfusion and infusion toward cerebral oxygen metabolism and cognitive recovery in the perioperative period of elderly knee replacement

**DOI:** 10.1186/1749-799X-9-8

**Published:** 2014-02-10

**Authors:** Changwei Wei, Yijin Yu, Yi Chen, Yuexia Wei, Xinli Ni

**Affiliations:** 1 Department of Anesthesiology, General Hospital of Ningxia Medical University, Yinchuan 750004, China

**Keywords:** Temperature, Cerebral extraction rate of oxygen, Jugular venous oxygen saturation, Cognitive function

## Abstract

**Objective:**

This study aims to observe the impact of the temperature of blood transfusion and infusion toward the perioperative cerebral oxygen metabolism and the postoperative cognitive recovery.

**Methods:**

Eighty patients of knee replacement under epidural and general anesthesia were randomly divided into warming blood transfusion and infusion (WBI) group (*n* = 40) and control group (*n* = 40). The changes of nasopharyngeal temperature, middle cerebral artery blood flow, CERO_2_, and SjVO_2_ of the two groups were recorded at each time point for the assessment of the postoperative overall quality of recovery and cognitive recovery situation.

**Results:**

The nasopharyngeal temperatures of the two groups at different time points after transfusion were significantly lower than that at T1, and there was a significant difference between the two groups (*P* < 0.05). The CERO_2_ values of the two groups at T3 were significantly higher than at T1, while the SjVO_2_ values were significantly decreased (*P* < 0.01).

**Conclusion:**

The WBI can significantly reduce the occurrence of the perioperative hypothermia, while it has no significant effect toward cerebral oxygen metabolism, postoperative overall recovery, and recovery of cognitive function.

## Introduction

Postoperative neuropsychological dysfunction is a common complication in elderly patients after joint replacement surgery. Some data indicated that [1] the incidence of postoperative cognitive dysfunction (POCD) in over 60-year-old patients was 25.8% 1 week after the major noncardiac surgery and 9.9% after 3 months. Transfusion is one of the risk factors of POCD [2]. A lot of blood transfusion could cause the occurrence of intraoperative hypothermia, especially for the elderly patients. According to the statistics [3], about 50%–90% of patients experienced the perioperative hypothermia, which is more prone toward the patients of knee replacement surgery, because the intraoperative low surrounding temperature environment and the cold flow from the air-conditioning would accelerate the heat loss of patients; the evaporation of skin disinfectant used would take the skin heat away, so the body temperature would drop; the massive infusion of cryogenic liquid would drop the body temperature of the patient. Sessler [4] found that when infused with 1,000 ml liquid at ambient temperature or cold-stored blood at 4°C per unit, the body temperature of an adult would drop 0.25°C; many studies [5-7] also revealed that the core temperature of 50%–70% patients would drop 1°C–3°C under general anesthesia state; when the core temperature of high-risk patient decreases 1.3°C, the occurrence probability of adverse cardiac events would increase twofold and the mechanism is still unclear. In addition, low body temperature could also cause hypokalemia, and hypokalemia is the important reason of ventricular tachycardia, ventricular fibrillation, and other arrhythmias diseases. The perioperative hypothermia would also increase the blood level of catecholamines in the circulation, resulting in the systemic vasoconstriction, which would then increase the risk of cardiovascular complications. Ozaki [8] found that toward the patients who suffered from perioperative hypothermia, the infection rate of the postoperative wound was 6.3 times that that with normal body temperature. Hypothermia has a double effect on the patients' coagulation; on one hand, it would make the circulating blood platelets decrease, decrease the platelet abilities of adhesion and aggregation, and reduce the activity of blood clotting factors, so that the bleeding time would be prolonged. A meta-analysis [9] pointed out that the intraoperative mild hypothermia would increase the blood loss of the patient by about 16% and the relative risk of transfusion by 22%. On the other hand, hypothermia would cause the peripheral vasoconstriction, venous stasis, and the reduction of tissue oxygen supply, which might further cause the deep vein thrombosis. The intraoperative hypothermia would cause the prolonged metabolism of anesthetics and muscle relaxants, and improve the organ solubility of volatile anesthetics, thus prolonging the recovery time from anesthesia. Therefore, in order to prevent the occurrence of hypothermia in patients, warming blood transfusion and infusion (WBI) is often clinically performed, and its benefits to the body have been reported in many relevant studies [10,11].

Low temperature has been identified with positive effects toward cerebral protection both in animal experiments and clinical practice. Ohnishi et al. [12] studied ten cases of coronary artery bypass surgery under cardiopulmonary bypass (CPB). The patients were randomly divided into normal-temperature CPB (NCPB) group and mild hypothermia CPB (HCPB) group. The results showed that the NCPB group could significantly inhibit the intraoperative cerebral hypoxia. Han et al. [13] observed the effects of mild hypothermia (30°C) and moderate hypothermia (26°C) on the cerebral oxygen metabolism in patients with mitral valve replacement. They found that during CPB, both mild hypothermia and moderate hypothermia could maintain the balance of cerebral oxygen metabolism, but further temperature did not guarantee the cerebral protective effect. Whether there was a cerebral oxygen metabolic imbalance after CPB still needed to be further studied. Whether or not WBI would be contrary to the concept of low-temperature brain protection, as well as whether or not WBI would have adverse effects toward neuropsychiatric recovery of the patients, the report is still few. Therefore, it is assumed that the intraoperative WBI might have a certain effect toward the perioperative cerebral oxygen metabolism and the postoperative cognitive function recovery in anesthetic elderly patients.

## Subjects and methods

### General information

The study was a prospective, randomized, single-blind, and controlled trial, and 80 patients who undergone single knee replacement surgery from Mar. 2012 to Feb. 2013 in the Department of Osteology, General Hospital of Ningxia Medical University were enrolled. The patients aged 60–75 years old, without gender limitation. The patients would be excluded if they were with a preoperative history of cerebrovascular disease and external injuries; episode of thrombotic event; coagulation disorders; knee infection complicated by systemic infection; educational attainment <7 years; severe visual or hearing impairment, unable to cooperate the completion of cognitive function test; secondary emergency surgery; and when they refuse to sign the informed consent. The patients were randomly divided into WBI group (*n* = 40) and control group (*n* = 40). WBI group used infusion fluid heating apparatus (Belmont FMS2000, Billerica, MA, USA) to warm the infusion blood and fluid up to 37°C. In the control group, the infusion blood and liquid were kept at room temperature (24°C) for 15–20 min before the direct infusion without warming treatment. Except for the researchers, the subjects, the surgeons, and the serum biochemical parameter detectors did not know the patient's grouping situation. This study was conducted in accordance with the declaration of Helsinki. This study was conducted with approval from the Ethics Committee of General Hospital of Ningxia Medical University. Written informed consent was obtained from all participants.

### Anesthesia method

All patients were given epidural and general anesthesia. Before anesthesia, the patient was intravenously injected with 10 mg dexamethasone and 0.5 mg atropine; the patient who had rapid heart rate would be given 0.3 mg scopolamine instead of atropine. Under the monitoring of electrocardiogram, saturation of pulse oxygen (SPO_2_), and continuous invasive arterial blood pressure (DRAGER, Lübeck, Germany), the patient had undergone L3-4 epidural catheterization, then intravenously administered with midazolam 0.05 mg/kg (Inverness, Xuzhou, China), fentanyl 2–3 μg/kg (Yichang blessing, Yichang, China), propofol 2 mg/kg (AstraZeneca, Luton, UK), and rocuronium 0.8–1.0 mg/kg (Merck & Co, Whitehouse Station, NJ, USA) for the general anesthesia induction, with mechanical ventilation by endotracheal intubation. The anesthesia status was maintained with the inhalation of 0.5%–1.5% isoflurane, and propofol was continuously infused at 2–4 mg/kg/h with micropump, with intermittent boluses of fentanyl (total 6–8 μg/kg). The respiratory parameters were set as follows: tidal volume as 8–10 ml/kg, respiratory rate as 10 to 12 times/min, maintaining the end-tidal carbon dioxide (ETCO_2_) at 35–45 mmHg (DRAGER, Germany). The intraoperative inspired-oxygen concentration was 100%, and the intraoperative blood pressure fluctuation should be ensured not to exceed 20% of baseline values. The fluid infusion of all patients started with 500 ml compound sodium chloride, followed by the infusion demand at crystal/gel ratio 2:1 and at the speed of 5–8 ml · kg^-1^ · h^-1^. Lower extremity tourniquet was used during the surgery, with the inflation pressure at 350–400 mmHg. The patients of the two groups were infused allogeneic or preoperative autologous blood through the peripheral vein 10 min before removing the tourniquet, with the speed as 15 ml/min. During the surgery, the amount of blood loss was calculated by the aspirator volume and gauze weighing. The postoperative wound drainage was recorded hourly; the drainage device could be removed until 48 h after the surgery. The postoperative patient-controlled epidural analgesia was performed, with 0.2% ropivacaine (AstraZeneca, UK) 2 ml/h for 72 h, followed by oral non-steroidal anti-inflammatory analgesics. The surgeons and the anesthesiologists of all patients were the same group of people.

### Monitoring methods and observation indicators

(1) Temperature monitoring: nasopharyngeal temperature probe (DRAGER) was used intraoperatively, monitoring the body temperature change at every time point. (2) Middle cerebral artery blood flow monitoring: the blood flow changes of all patients were measured in bilateral middle cerebral artery (MCA) through the temporal window at preoperatively (T0), after anesthesia and before transfusion (T1), immediately after blood transfusion (T2), 30 min after transfusion (T3), 1 h after transfusion (T4), and 2 h after transfusion (T5), with Companion 2021-III type Transcranial Doppler instrument (EME, Ingolstadt, Germany). The probe frequency was 2 MHz, with the depth set as 46–60 mm. The contralateral MCA blood flow of the patients whose temporal window was of poor sound transmission was then measured through the eye window, with the depth set as 80–92mm. The neck compression test should be performed to confirm the blood vessels detected. The MCA blood flow speed peaks in systolic phase (Vs) and in diastolic phase (Vd) were recorded for the calculation of the resistance index (RI) [RI = (Vs - Vd)/Vs]. (3) Jugular venous oxygen saturation (SjVO_2_) and cerebral extraction rate of oxygen (CERO_2_) monitoring: the patient was in supine position, with head in neutral line and parallel to the bedside. After local anesthesia, the internal jugular vein paracentesis was performed from the spot slightly outside the carotid pulse and parallel to the lower edge of thyroid cartilage, and along the ipsilateral external ear direction. After the success of paracentesis, 5F bi-cavity deep vein catheter was directed inside, with the catheter tip in the equivalent position of external ear canal (i.e., of internal jugular bulbar zone). The catheter was sealed with heparin saline, and the radial arterial blood and jugular bulbar blood were extracted synchronously at T0–T5 for the blood gas analysis, respectively; the SjVO_2_ value was monitored and CERO_2_ calculated. (4) Assessment of the postoperative recovery quality and the cognitive function: the Post-operative Quality Recovery Scale (PQRS) of all subjects were tested preoperatively, 15 and 40 min after extubation, postoperative 1 and 3 days, respectively [14] PQRS is a comprehensive scale for the real-time assessment of the recovery quality after general anesthesia, developed by a number of international anesthesiological experts. It is mainly focused on five indicators, namely the physiological functions, noxious stimuli, emotion, daily living activities, and cognitive function recovery, assessing hierarchically at different postoperative time points and describing comprehensively the overall recovery situation. If the postoperative score of each item is equal to or greater than the baseline value, this item would be considered as restored fully; if the value is below the baseline level, the item would be deemed as not recovered. If one of the five indicators is lower than the baseline value, the situation would be considered as not overall recovered.

### Statistical analysis

All statistical processing was performed by SPSS16.0 software. The measurement data were expressed as mean ± SD, with the incidence rate represented by percent. The intergroup comparison was performed using two independent samples *t* test, while the intragroup comparison used the analysis of variance of the repeated measurement, the count data used chi-square test, with *P* < 0.05 considered as statistically significant.

## Results

### Comparison of general data between two groups

There were no significant differences in age, sex, height, weight, operation time, extubation time, tourniquet time, blood loss, blood transfusion, or preoperative PQRS baseline value between the two groups (Table 1).

**Table 1:** **Comparison of the general information of the two groups**$\left(\bar{\chi }\pm s,n=40\right)$

**Item**	**WBI**	**Control**	***t***	***P***
Age (years)	53.68 ± 10.97	59.00 ± 8.73	1.8437	>0.05
Height (cm)	163.37 ± 6.95	163.42 ± 6.68	0.0345	>0.50
Weight (kg)	68.00 ± 15.06	71.89 ± 14.76	1.1680	>0.20
Years of education (years)	11.84 ± 2.03	11.37 ± 2.31	0.9666	>0.10
Operation time (min)	151.21 ± 21.76	147.84 ± 23.43	0.6666	>0.50
Tourniquet time (min)	91.42 ± 14.32	89.33 ± 13.26	0.6773	>0.50
Extubation time (min)	35.21 ± 6.45	37.28 ± 8.42	0.4532	>0.50
Blood transfusion (ml)	521.05 ± 127.27	536.84 ± 146.10	0.5154	>0.50
Blood loss (ml)	788.68 ± 133.38	810.05 ± 157.43	0.6550	>0.50
Total infusion (ml)	2,973.68 ± 625.25	2,971.05 ± 551.09	0.0199	>0.50
Total loss (ml)	1,646.84 ± 401.64	1,717.90 ± 397.11	0.7957	>0.40

### Comparison of nasopharyngeal temperature between two groups

The nasopharyngeal temperatures of the two groups at various postoperative time points were significantly lower than T1. There were significant differences of nasopharyngeal temperatures at each time point between the two groups (*P* < 0.01, Table 2).

**Table 2:** **Impacts of different transfusion temperatures on nasopharyngeal temperature (°C), CERO**_**2**_**(%), SjVO**_**2**_**(%)**$\left(\bar{\chi }\pm s,n=40\right)$

**Item**	**Group**	**T0**	**T1**	**T2**	**T3**	**T4**	**T5**
Nasopharyngeal temperature	WBI	36.45 ± 0.21	36.02 ± 0.43	35.83 ± 0.47^ac^	35.68 ± 0.58^ac^	35.98 ± 0.47^a^	36.09 ± 0.40^b^
	Control	36.43 ± 0.21	35.98 ± 0.45	35.22 ± 0.38^c^	35.08 ± 0.42^c^	35.69 ± 0.32^c^	35.90 ± 0.33
CERO_2_	WBI	43.95 ± 12.89	38.60 ± 13.68	41.39 ± 11.60	48.32 ± 10.37^c^	42.92 ± 10.08	42.69 ± 10.67
	Control	45.97 ± 11.70	40.60 ± 16.17	46.04 ± 12.88	49.16 ± 14.53^c^	46.13 ± 15.08	45.58 ± 14.52
SjvO_2_	WBI	53.12 ± 8.67	62.62 ± 11.67	55.68 ± 8.25	48.05 ± 10.98^c^	54.75 ± 6.12	54.80 ± 4.96
	Control	51.75 ± 8.14	60.02 ± 13.43	54.10 ± 6.23	46.85 ± 7.42^c^	54.22 ± 7.39	53.98 ± 6.42

Compared with control group, ^a^*P* < 0.01, ^b^*P* < 0.05; compared with T1, ^c^*P* < 0.01.

### Comparison of middle cerebral artery blood flow between two groups

In the two groups, the Vs at T2 and T3 were significantly lower than at T1 (*P* < 0.05), but the difference between the two groups at all time points was not statistically significant (*P* > 0.05). The RI of control group at T2 was significantly higher than at T1 (*P* < 0.05), while the difference between the two groups at different time points was not statistically significant (*P* > 0.05, Table 3).

**Table 3:** **Impacts of different transfusion temperature on perioperative Vs (cm/s), Vd (cm/s), and RI**$\left(\bar{\chi }\pm s,n=40\right)$

**Item**	**Group**	**T0**	**T1**	**T2**	**T3**	**T4**	**T5**
Vs	WBI	90.72 ± 5.29	89.76 ± 5.41	89.34 ± 5.41^a^	89.08 ± 5.61^b^	89.71 ± 5.65	90.35 ± 4.91
	Control	90.74 ± 4.57	89.57 ± 5.63	88.54 ± 5.59^a^	88.04 ± 4.44^b^	89.14 ± 4.43	90.16 ± 5.30
Vd	WBI	43.72 ± 3.16	43.20 ± 3.10	42.83 ± 2.93	42.68 ± 3.13	43.03 ± 3.39	43.71 ± 3.19
	Control	43.33 ± 2.95	42.92 ± 3.58	41.88 ± 3.35	41.35 ± 3.28	42.32 ± 3.27	43.30 ± 3.45
RI	WBI	0.52 ± 0.02	0.52 ± 0.04	0.52 ± 0.03	0.53 ± 0.04	0.52 ± 0.03	0.51 ± 0.02
	Control	0.52 ± 0.03	0.52 ± 0.03	0.53 ± 0.05^b^	0.52 ± 0.04	0.51 ± 0.02	0.51 ± 0.03

Compared with T1, ^a^*P* < 0.01, ^b^*P* < 0.05.

### Comparisons of CERO_2_ and SjVO_2_ between two groups

The CERO_2_ of the two groups at T3 were significantly higher than at T1, while the SjVO_2_ significantly reduced (*P* < 0.01). However, the CERO_2_ and SjVO_2_ at each time point had no statistically significant difference between the two groups (*P* > 0.05, Table 2).

### Comparisons of overall recovery quality and cognitive function restoration between two groups

The overall quality of recovery and restoration of cognitive function at postoperative 15 min, 40 min, 1 day, and 3 days showed no significant difference between the two groups (*P* > 0.05, Table 4).

**Table 4:** **Impacts of different transfusion temperature on the unrecovered rates of postoperative overall and cognitive function (%,** ***n*** **= 40)**

**Item**	**Group**	**15 min after extubation**	**40 min after extubation**	**Postoperative 1 day**	**Postoperative 3 days**
Overall unrecovered rate	WBI	100	100	25	19
	Control	100	100	32	23
Unrecovered cognitive rate	WBI	87.5	62.5	52.5	40.0
	Control	77.5	60.0	45.0	35.0

## Discussion

A large number of experimental studies and clinical practice have proven that moderate hypothermia has certain neuroprotective effect toward the ischemic brain injury; however, clinical practice found that low temperature (<36°C) could cause chills, coagulation disorders, cardiovascular dysfunction, metabolic abnormalities, etc. [15,16]. Low temperature would also inhibit the vasoconstriction and the immune system, increasing perioperative incidence of wound infections. The total knee replacement is mostly performed toward elderly patients, the surgical wound is large, the blood oozing is much more, and the blood transfusion would often lead to the perioperative hypothermia in patients. Therefore, most scholars advocate that the perioperative body temperature management should be strengthened. The current clinical practice would prevent the occurrence of intraoperative hypothermia through controlling the room temperature, using heat preservation blanket, enhancing the body surface coverage, using heating infusion devices, etc. [10,17]. However, it should be given attention that some studies had found that toward the elderly patients of total knee replacement surgery, the perioperative warming or keeping warmth would result in the incidence of cognitive dysfunction on the postoperative fourth day increased from 3.2% to 14.9% [18]. In the present study, we observed and compared the impact of warming and non-warming blood transfusion and infusion on the perioperative cerebral oxygen metabolism, postoperative quality of recovery, and cognitive recovery characteristics in knee replacement surgery, and we found that the nasopharyngeal temperatures of the two groups dropped at each time point after the transfusion, while the nasopharyngeal temperatures of warming blood transfusion group were significantly higher than the control group at all time points after the transfusion, which might be due to the knee replacement surgery that was mostly performed on elderly patients, whose thermoregulatory function reduced, so the self-regulating capacity toward the perioperative changes of body temperature was poor; additionally, because the general anesthesia drugs would inhibit the thermoregulatory center and enlarge the blood vessels, which exacerbated the loss of body heat, hypothermia would be much easier to happen. The intraoperative WBI could reduce the heat loss and effectively prevent the shivering in the recovery of senile knee replacement surgery.

In this study, transcranial Doppler instrument was used to monitor the indicators which could reflect the cerebral blood flow velocity (CBFV) in the middle cerebral artery, including Vs, Vd, and Vm (mean blood flow velocity), among which Vs represented the highest blood flow velocity in systolic period, reflecting the highest MCA flow velocity during entire cardiac cycle; Vd represented the highest MCA blood flow velocity at the end of cardiac cycle, reflecting the vascular resistance; RI mainly reflected the cerebrovascular resistance condition. If there was no significant change in vessel diameter, the above indicators would increase with the CBFV, and the cerebral blood flow (CBF) would also increase; but when the vascular spasm or strong contraction happened, through CBFV would increase, CBF might have no change or even decrease; while the vasodilation at this time would make CBFV reduce, CBF would increase or exhibit no change. In this study, the Vs of the two groups decreased immediately after the transfusion and still decreased 30 min after the transfusion; it might probably be because of the relief of tourniquet pressure, the lower limb blood vessels could dilate, causing the CBF slow down. The intergroup comparison exhibited no significant difference, indicating that compared with the control group, the WBI had no effect on cerebral blood flow velocity of elderly knee replacement patients; it might partially be because the transfusion volume in this study was relatively small and the degree of hypothermia did not break 35°C.

In this study, CERO_2_ and SjVO_2_ were simultaneously monitored for the comprehension of the balance of perioperative cerebral oxygen supply and demand. Under normal circumstances, when the CBF is constant, CERO_2_ would reduce as the temperature decreases, while SjVO_2_ would relatively increase. In this study, the CERO_2_ of the two groups 30 min after transfusion were significantly higher than those before the transfusion, and SjVO2 were significantly lower than those after anesthesia while before transfusion, but they were still within the normal ranges. We considered the above situation might be because of the insufficient cerebral blood supply, which was induced by the acute bleeding in short time after the tourniquet was released. Because the major organ reservation and the compensatory function of elderly patients significantly reduce, the biotransformation and elimination rates of liver and kidney toward the narcotic drug would significantly prolong, resulting in the poor quality of postoperative recovery, even the occurrence of POCD [19]. Although the pathological mechanism of POCD still remains unclear, the patient's own illness situation, anesthesia technology, surgical trauma, and perioperative physiological status would be closely related with the occurrence of POCD. Hong [20] indicated that the monitoring of regional cerebral oxygen saturation could not predict the incidence of postoperative cognitive dysfunction in cardiac valve replacement patients, but it could be used as the indicator to evaluate the body tissues and organ perfusion and the patients' prognosis.

Although studies have shown [18] that the intraoperative tympanic temperature above 36°C was an independent risk factor of POCD, there was also study considered that [21] the intraoperative body temperature and anesthesia duration would not affect the postoperative cognitive function recovery. PQRS is a new scale developed for the assessing the quality of postoperative recovery after general anesthesia; this study applied PQRS scale and found that there were no statistically significant differences in the overall quality of recovery of the two groups 15 min, 40 min, 1 day, and 3 days after extubation. Royse et al. [14] reported that, at the postoperative 3 days, only 33.5% patients with total knee arthroplasty (TKA) had recovery of cognitive function. There might be many reasons leading to occurrence of false positive case. Later, the evaluation method was adjusted, and the results showed that, at the postoperative 3 days, the proportion of patients with cognitive function recovery increased from original 33.5% to 86.4%. This indicated that, at the postoperative 3 days, most patients had recovery of cognitive function. In addition, in this study, the hospitalization duration after TKA was relatively short. Therefore, the latest observation time was selected as the postoperative 3 days, for obtaining more useful data. At the postoperative 3 days, although the rates of unrecovered cognitive function of the two groups were 40% and 35% on the postoperative 3 days, respectively, the difference was not statistically significant, indicating that toward the elderly patients after single knee replacement surgery, the intraoperative WBI had no significant effect on the cognition and overall recovery. But whether or not the large amount of transfusion would need warming and whether or not the warming would have positive impact on the postoperative recovery still remained to be clarified by further large randomized controlled multi-center clinical studies.

The objects of study are patients with knee replacement surgery, and the intraoperative blood transfusion amount is less than normal. In addition, the follow-up of postoperative overall recovery quality and cognitive function restoration is performed just for three postoperative days, without long-term follow-up (6 months or 1 year). These are the limitations of this study. Next, based on results of this study, the research objects should be patients with large blood transfusion in surgery. The change of perioperative cerebral oxygen metabolism and overall recovery quality and cognitive function restoration in one postoperative year will be observed. The objective is to decide performing WBI or not, and guide clinical reasonable blood transfusion.

It is found that the blood transfusion is a risk factor for postoperative early cognitive dysfunction in elderly patients. Whether intraoperative infusion of preoperative autologous blood or allogeneic blood will influence the postoperative cognitive function to a certain degree, and this mainly depends on the amount of infused blood. More than 1,000 ml of infused blood is considered as the independent risk factor for postoperative delirium [22]. In this study, the autologous blood collection was performed in most patients for a week before operation. For most patients, the intraoperative infusion of autologous blood can meet the requirement of the body. On one hand, the objective of infusion of allogeneic blood was to prevent postoperative dominant and recessive anemia. It is found that the proportion of patients with allogeneic blood transfusion after TKA is as high as 69% [23]. On the other hand, infusion of allogeneic blood could enhance the reserve function in elderly patients. This study has not strictly discriminated the allogeneic blood and preoperative autologous blood. The reason is that our research focus is to observe the impact of warming blood transfusion and infusion toward cerebral oxygen metabolism and cognitive recovery in the perioperative period of elderly knee replacement. This study is a prospective, randomized, single-blind, and controlled trial, and whether preoperative autologous blood or allogeneic blood does not influence the results. In summary, toward the elderly patients with single knee replacement surgery, the intraoperative WBI had no significant effect on the perioperative cerebral oxygen metabolism and the cognitive function recovery.

## Conclusion

For elderly patients treated by single knee replacement surgery, intraoperative WBI can reduce the occurrence of perioperative hypothermia, which is not contrary to the concept of cerebral protection by low temperature. The intraoperative WBI has no significant effect on the perioperative cerebral oxygen metabolism or cognitive function recovery. In addition, sometimes the WBI device cannot be used, for saving medical resources and reducing expense of patients and medical burden of society.

## Abbreviations

POCD: postoperative cognitive dysfunction; RI: resistance index.

## Competing interests

The authors declare that they have no competing interest.

## Authors’ contributions

XN and CW designed this paper and performed critical revision of the manuscript. YY and YC performed data collection. YW analyzed the date. XN wrote the manuscript. All authors read and approved the final manuscript.
